# Protection against Marburg Virus and Sudan Virus in NHP by an Adenovector-Based Trivalent Vaccine Regimen Is Correlated to Humoral Immune Response Levels

**DOI:** 10.3390/vaccines10081263

**Published:** 2022-08-05

**Authors:** Machteld M. Tiemessen, Laura Solforosi, Liesbeth Dekking, Dominika Czapska-Casey, Jan Serroyen, Nancy J. Sullivan, Ariane Volkmann, Maria Grazia Pau, Benoit Callendret, Hanneke Schuitemaker, Kerstin Luhn, Roland Zahn, Ramon Roozendaal

**Affiliations:** 1 Janssen Vaccines & Prevention B.V., Archimedesweg 6, 2333 CN Leiden, The Netherlands; 2 Vaccine Research Center, National Institute of Allergy and Infectious Diseases, National Institutes of Health, Bethesda, MD 20892, USA; 3 Bavarian Nordic GmbH, Fraunhoferstrasse 13, D-82152 Martinsried, Germany

**Keywords:** Ebola virus, Marburg virus, Sudan virus, filovirus, vaccine, Ad26, non-human primate (NHP), glycoprotein (GP)–binding antibody

## Abstract

The Marburg virus (MARV) and Sudan virus (SUDV) belong to the filovirus family. The sporadic human outbreaks occur mostly in Africa and are characterized by an aggressive disease course with high mortality. The first case of Marburg virus disease in Guinea in 2021, together with the increased frequency of outbreaks of Ebola virus (EBOV), which is also a filovirus, accelerated the interest in potential prophylactic vaccine solutions against multiple filoviruses. We previously tested a two-dose heterologous vaccine regimen (Ad26.Filo, MVA-BN-Filo) in non-human primates (NHP) and showed a fully protective immune response against both SUDV and MARV in addition to the already-reported protective effect against EBOV. The vaccine-induced glycoprotein (GP)-binding antibody levels appear to be good predictors of the NHP challenge outcome as indicated by the correlation between antibody levels and survival outcome as well as the high discriminatory capacity of the logistic model. Moreover, the elicited GP-specific binding antibody response against EBOV, SUDV, and MARV remains stable for more than 1 year. Overall, the NHP data indicate that the Ad26.Filo, MVA-BN-Filo regimen may be a good candidate for a prophylactic vaccination strategy in regions at high risk of filovirus outbreaks.

## 1. Introduction

Marburg virus (MARV) and Sudan virus (SUDV) are single-strand negative-sense RNA viruses belonging to the family of filoviruses. Human outbreaks of MARV and SUDV predominantly occur in sub-Saharan Africa, are sporadic by nature, and are characterized by their aggressive disease course as defined by high mortality. The recent first case of Marburg virus disease in Guinea in 2021 [1], together with the increase in frequency of outbreaks of Ebola virus disease (EVD) caused by another filovirus, Ebola virus (EBOV), sharpened the interest in potential prophylactic vaccine solutions [2].

The increase in frequency of EVD outbreaks is seen in specific geographical locations, such as the Democratic Republic of Congo (DRC), where the re-occurrence of EVD outbreaks is most likely caused by zoonotic transmission events from animals to humans [3]. In addition, other recent EVD outbreaks are attributed to EBOV that persisted in EVD survivors [4]. Although the presence of EBOV is undetected in the serum of EVD survivors, the presence of virus has been demonstrated in bodily fluids, such as semen and breast milk, and at immuno-privileged sites, such as the eyes, testes, and brain, for up to 5 years [5]. This persistence of the virus in survivors may cause outbreaks at unpredictable moments in time [6]. While sporadic outbreaks at unpredictable locations require a coordinated emergency response at the time of an outbreak, viral persistence-derived transmission of EBOV or zoonotic transfer in high risk areas (e.g., Mbandaka, the DRC) can be interrupted by prophylactic vaccination. In a prophylactic setting, it would be beneficial to also confer protection against additional members of the filovirus family, MARV and SUDV, which are also reported to be caused by animal-to-human transmission [7,8]. Accelerated vaccine development in response to the largest EBOV outbreak in history, with more than 28,646 cases [9,10], and the availability of a large clinical safety database generated from early-generation Ebola glycoprotein (GP) vaccines based on DNA and adenovirus vectors, resulted in the regulatory approval of two EBOV vaccines. The first, Ervebo^®^ (Merck, Whitehouse Station, NJ, USA), licensed by the U.S. Food and Drug Administration, is a replication-competent recombinant vesicular stomatitis virus (rVSV)–based vaccine, encoding the GP of EBOV Kikwit [11,12]. The second Ebola vaccine, the Zabdeno^®^, Mvabea^®^ regimen (Janssen Vaccines & Prevention, Leiden, The Netherlands), licensed by the European Medical Agency, is a two-dose regimen consisting of a replication-incompetent adenoviral vector serotype 26 (Ad26) encoding the EBOV Mayinga GP (Ad26.ZEBOV) as a first dose and a recombinant, non-replicating modified vaccinia Ankara–vectored vaccine encoding the EBOV Mayinga, SUDV Gulu, and MARV Musoke GPs and the nucleoprotein of the Tai Forest virus (MVA-BN-Filo) as a second dose.

Initial studies on the Ad26.ZEBOV, MVA-BN-Filo vaccine regimen demonstrated the regimen to be immunogenic and efficacious against EBOV Kikwit infection in non-human primates (NHP) [13]. Further analysis of the vaccine-induced immune responses showed both humoral and cellular responses as measured by high levels of EBOV GP–specific binding and neutralizing antibodies, and the presence of GP-specific T cell responses [14]. Of all measured immunological markers, the EBOV GP–specific binding antibody concentration appeared to be the best predictor of survival in NHP [14]. Importantly, the Zabdeno, Mvabea regimen was well tolerated in humans and elicited a strong GP-specific binding antibody response [15,16]. Based on the immune response levels in humans, it was inferred that the Zabdeno, Mvabea regimen would have a clear protective effect in humans [17]. Such an approach is also especially relevant for the development of MARV and SUDV vaccines, where limited size and frequency of the outbreaks pose an even greater challenge for obtaining human effectiveness data.

We previously explored the feasibility of providing protection against multiple filovirus species with a multivalent vaccine [13,18]. Individual adenoviral vectors (serotypes Ad26 and Ad35) encoding the EBOV Mayinga GP, SUDV Gulu GP, or MARV Angola GP (the combination of these three vectors in a 1:1:1 ratio will be further referred to as Ad26.Filo and Ad35.Filo) were generated. Prophylactic vaccination with Ad26.Filo has been investigated in NHP studies in a homologous vaccine regimen (Ad26.Filo, Ad26.Filo) as well as a heterologous vaccine regimen with three Ad35 vectors encoding the same GP of EBOV, SUDV, and MARV as a second dose (Ad26.Filo, Ad35.Filo). Both the homologous (Ad26, Ad26) and heterologous (Ad26, Ad35) vaccine regimens elicited a fully protective immune response in NHP against MARV Angola challenge, while the protection against SUDV Gulu challenge and EBOV Kikwit was partial (75% and 50%, respectively). Importantly, full protection against the EBOV Kikwit challenge could be achieved when the Ad26.Filo vector was combined with the MVA-BN-Filo vector as dose 2 [13].

In the current studies, we provide evidence that a two-dose regimen, consisting of Ad26.Filo followed by MVA-BN-Filo with an 8-week interval, provided 100% protection against SUDV Gulu and MARV Angola challenge in NHP. Moreover, there was a strong correlation between the level of GP-specific binding antibodies and survival outcome with a high discriminatory capacity. The vaccine-induced humoral response against SUDV, EBOV, and MARV reached stable GP-binding antibody levels that persisted for >1 year after immunization.

## 2. Materials and Methods

### 2.1. Viral Vaccines

Replication-incompetent, E1/E3-deleted recombinant adenoviral vectors based on Ad26 and Ad35 were engineered using the AdVac^®^ system (Janssen Vaccines & Prevention B.V., Leiden, The Netherlands) with the humanized GP DNA sequences for EBOV Mayinga (Ad26.ZEBOV), SUDV Gulu (Ad26.SUDV), and MARV Angola (Ad26.MARVA). The combination of these three Ad26 vectors in a 1:1:1 ratio will be further referred to as Ad26.Filo; when the Ad35 vectors are used, the combination will be referred to as Ad35.Filo. Rescue and manufacturing of the vectors were performed in the complementing cell line PER.C6^®^ as previously described [18]. The Ad5 vector containing the GP DNA sequence of MARV Angola was described by Callendret et al. in 2018 [13]. MVA-BN-Filo (Bavarian Nordic, Hellerup, Denmark) is a recombinant, modified vaccinia Ankara-vectored vaccine, non-replicating in human cells, encoding the EBOV Mayinga, SUDV Gulu, and MARV Musoke GPs as well as the nucleoprotein of the Tai Forest virus. Adenovirus vaccines were given at 1.2 × 10^11^ viral particles (vp; 4 × 10^10^ vp/vector) and the MVA-BN-Filo vector at a dose of 5 × 10^8^ infectious units (infU).

### 2.2. Ethics Statement

All animal research protocols were approved by either the Texas Biomedical Research Institute (TBRI, San Antonio, TX, USA), Bioqual Inc. (Bioqual, Rockville, MD, USA), Charles River Laboratories (CRL, Reno, NV, USA), the National Institutes of Health (NIH) Vaccine Research Center (Bethesda, MD, USA), or the United States Army Medical Research Institute for Infectious Disease (USAMRIID, Fort Detrick, MD, USA) Institutional Animal Care and Use Committee in compliance with the Animal Welfare Act, Public Health Service Policy on Humane Care and Use of Laboratory Animals, and other federal statutes and regulations relating to animals and experiments involving animals. Vaccination phases were conducted at Bioqual, TBRI, or CRL. Challenge phases were performed at TBRI or USAMRIID.

Adult cynomolgus monkeys of Mauritian (SUDV challenge study) and Vietnamese (MARV challenge study) origin were used in the studies with males and females. There was no obvious contribution of macaque origin to either vaccine immunogenicity or challenge outcome, although this was not formally tested. Euthanasia was performed in accordance with the recommended method of the Panel on Euthanasia of the American Veterinary Medical Association. Animals were sedated prior to administration of an overdose of pentobarbital sodium via the intracardiac route.

Vaccinations were given intramuscularly (i.m.) in the left posterior thigh (quadriceps) or bilateral deltoid at the indicated dosages and time points in the study. All animals were sedated with 10 mg/kg ketamine (and 1 mg/kg acepromazine at the Bioqual facilities) i.m. prior to immunizations.

### 2.3. Filovirus Challenge Material and Animal Challenge

Immunized animals were transferred from biosafety level (BSL)-2 to BSL-4 facilities (USAMRIID for the SUDV and MARV challenge study and TBRI for the MARV challenge study) and acclimatized for 7 to 8 days before challenge. Challenge stocks were deep sequenced to confirm wild-type identity of the viruses, and all stocks were confirmed as endotoxin-free before usage. For MARV, a second passage MARV Angola challenge strain (Lot No. 201206191, originally obtained from the 2005 outbreak [19]) was obtained from Dr. Tom Ksiazek (at the National Institute of Allergy and Infectious Diseases World Reference Center for Emerging Viruses and Arboviruses at the University of Texas Medical Branch Health Galveston National Laboratory) in 2012 and propagated in Vero E6 cells. The third passage was used as challenge material in this study at TBRI. For SUDV, the challenge material was derived from a third passage in Vero E6 cells of a clinical specimen from an outbreak occurring in Uganda in 2000 [20]. Animals were sedated prior to receiving the challenge dose (1000 plaque-forming units [pfu]) i.m. in the right deltoid arm muscle (0.5 mL volume).

Animals were monitored for health status twice daily and more frequently as clinical signs warranted. A clinical scoring system was used to monitor and report clinical signs of disease with the use of a clinical observation sheet. At USAMRIID, the scoring system assessed activity, responsiveness to external stimuli, posture, and signs of respiratory distress. A score of 3 triggered a blood draw to measure secondary euthanasia criteria. A score of 4, the maximum score, indicated that an animal met the primary criteria for euthanasia. At TBRI, animals were scored in 13 categories (graded scale each) to monitor their health. Categories consisted of monitoring of weight loss, temperature change, responsiveness, hair coat, respiration, petechiae, bleeding, nasal discharge, food eaten, food enrichment intake, stool, fluid intake, and dehydration. If a score was ≥5, the animal was reported to the veterinarian on site for further assessment. If a total score of ≥15 was reached, the animal was considered terminally ill and euthanized.

### 2.4. Filovirus Glycoprotein Enzyme-Linked Immunosorbent Assay (ELISA) and Filovirus GP–Reactive Interferon Gamma (IFN-γ) Producing T-Cell ELISpot

All GP-specific ELISA and ELISpot assays were performed with NHP sera and NHP peripheral blood mononuclear cells (PBMC), respectively, at TBRI and have been described previously [13]. The EBOV and MARV GP–specific ELISA of the sera of NHP, immunized and followed up for more than 1 year, was performed at Battelle Biomedical Research Center (Columbus, OH, USA) using a previously described protocol [21]. The ELISA for the single-shot Ad26-MARV study was performed as described previously [22].

### 2.5. Virus Neutralization Assays

The virus neutralization assays were performed at Monogram Biosciences (San Francisco, CA, USA). NHP serum samples were subject to EBOV and/or SUDV and/or MARV neutralization assay (PhenoSense^®^ Pseudovirus Neutralizing Antibody Assay [Monogram Biosciences, San Francisco, CA, USA]). The assays have been qualified (SUDV and MARV virus-neutralizing antibody [VNA]) or validated (in case of EBOV VNA). The PhenoSense^®^ Pseudovirus Neutralizing Antibody Assay is a single virus replication cycle assay, which is an adaptation of the Monogram PhenoSense^®^ HIV Neutralizing Antibody Assay and Entry/Tropism Assay. A replication-defective retroviral vector containing a luciferase gene was co-transfected into human embryonic kidney 293 (HEK 293) cell cultures along with an expression vector containing Ebola or Marburg virus (EBOV Makona 2014, SUDV Gulu, or MARV Angola) envelope GP sequences. Pseudovirus stocks were harvested and incubated with serial dilutions of serum samples for 18 h and then used to infect HEK 293 cell cultures. Each serum sample was serially diluted four-fold to yield 10 assay conditions (starting dilution of 1:40). The ability of antibody in the serum to neutralize EBOV, MARV, or SUDV infectivity was assessed by measuring luciferase activity ~72 h after viral inoculation as compared to a control infection using an amphotropic murine leukemia virus (aMLV) envelope-pseudotyped virus. Neutralization titers are expressed as the reciprocal of the serum dilution that inhibited the virus infection by 50%.

### 2.6. Statistical Methods

In general, there was no statistical analysis plan associated with the individual studies. Logistic regression models were used to explore the association between GP-specific binding antibody concentration and survival outcome after challenge. The capacity of the logistic regression models to discriminate between survivors and non-survivors was assessed with the area under the curve (AUC) of the receiver operating characteristic (ROC) curve. GP-specific binding antibody concentrations measured 1 week prior to challenge were included from several independent studies per virus (as indicated in Supplementary Tables S1 and S2). All statistical analyses were performed in R version 4.1.1 (Vienna, Austria) [23]. Differences in vaccine-induced levels of GP-specific binding antibody responses between regimens were assessed by non-parametric Mann–Whitney U tests.

## 3. Results

### 3.1. Immunogenicity and Protective Efficacy against SUDV Challenge

Twenty cynomolgus macaques were divided into four groups of 5 animals each (Table 1).

**Figure 1:**
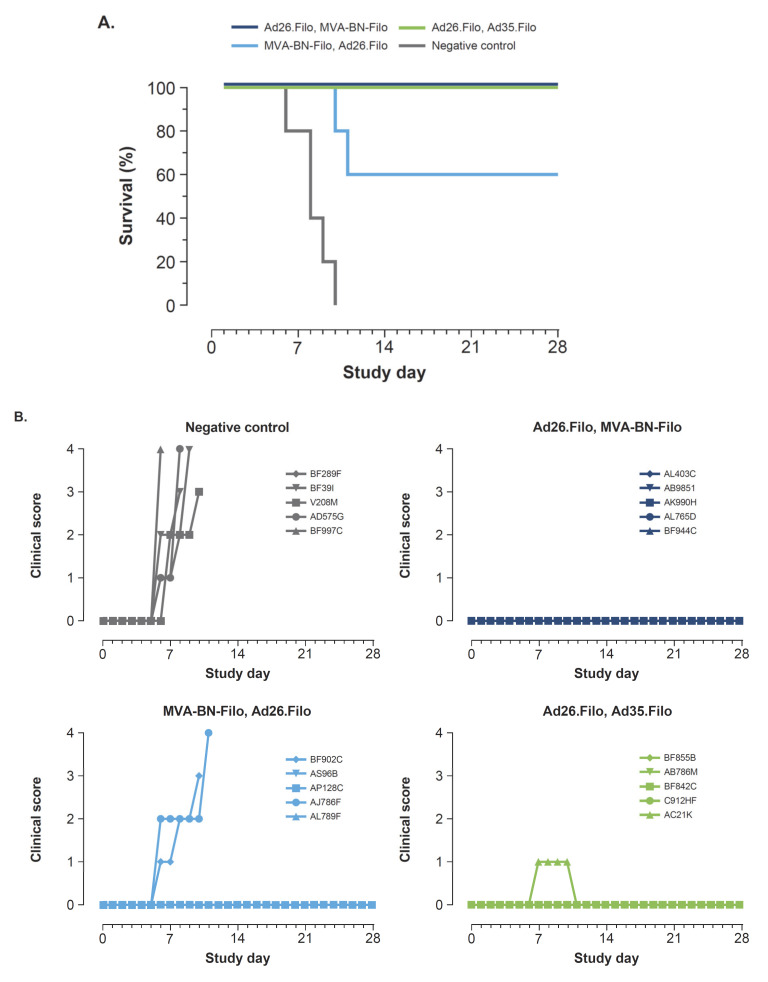

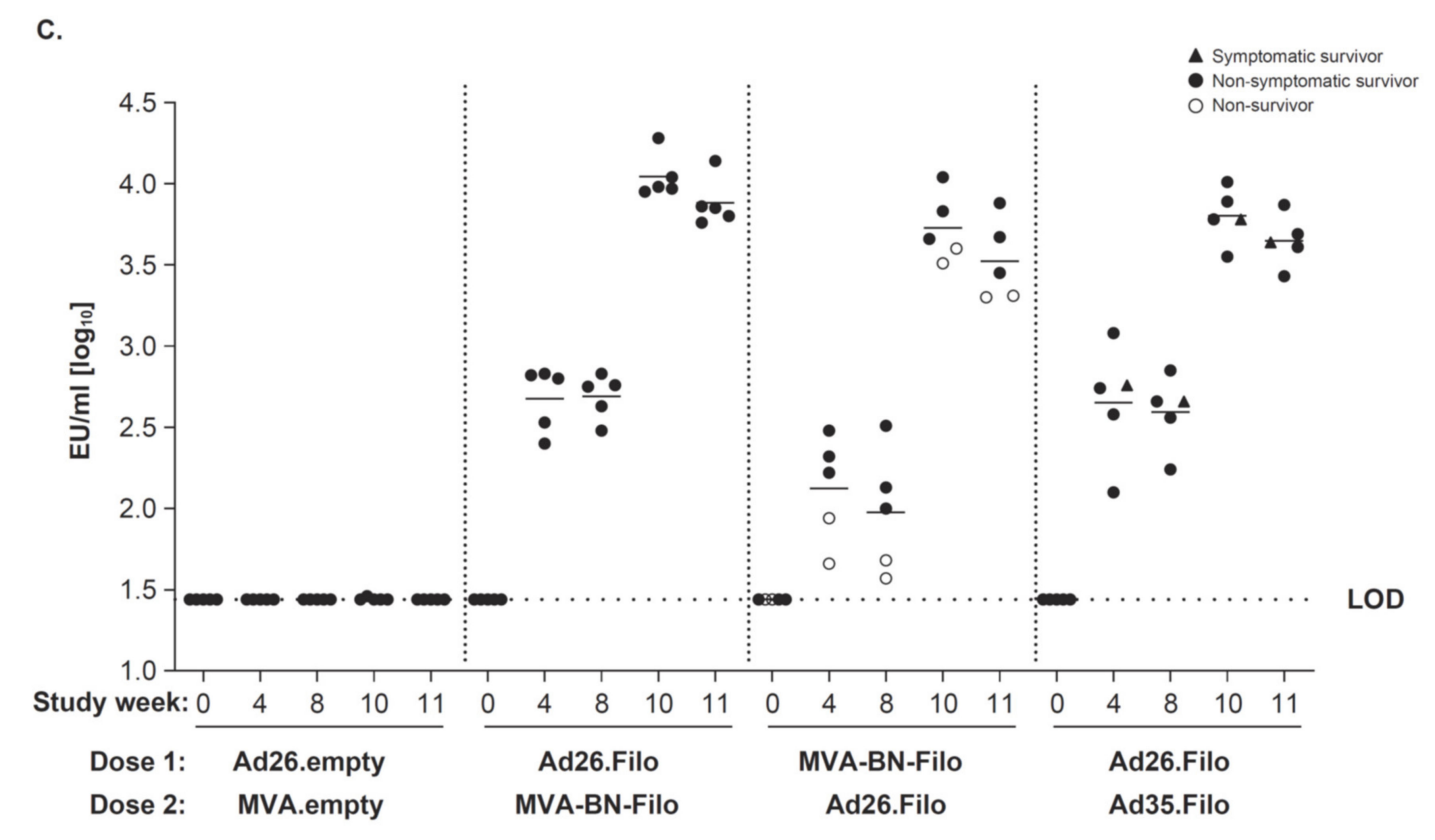
A trivalent two-dose heterologous regimen fully protects against lethal SUDV challenge. (**A**–**C**) Twenty cynomolgus macaques were immunized i.m. with Ad26.Filo as dose 1 at week 0 and MVA-BN-Filo or Ad35.Filo as dose 2 at week 8, or with MVA-BN-Filo as dose 1 and Ad26.Filo as dose 2, or empty Ad26 and empty MVA-BN vector (n = 5 per group). Adenovirus vaccines were given at 1.2 × 10^11^ vp (4 × 10^10^ vp/vector) and the MVA-BN-Filo vector at a dose of 5 × 10^8^ infU. A challenge with 1000 pfu SUDV Gulu was given i.m. 4 weeks post-dose 2. (**A**) Kaplan-Meier representation of survival. (**B**) Clinical scoring, according to the scale of USAMRIID, of individual animals after lethal challenge. (**C**) Humoral immune response over time measured by SUDV GP–specific ELISA. Each symbol represents an individual NHP at the indicated time point, with the closed circles representing the non-symptomatic survivors, open circles the non-survivors, and triangles the symptomatic survivors (symptomatic is defined as clinical score > 0). The black horizontal line indicates means per group, and the dashed horizontal line represents the LOD. Ad26, adenoviral vector serotype 26; MVA, modified vaccinia Ankara; Ad35, adenoviral vector serotype 35; ELISA, enzyme-linked immunosorbent assay; EU, ELISA unit; LOD, limit of detection; SUDV, Sudan virus; i.m., intramuscularly; vp, viral particles; infU, infectious unit; pfu, plaque-forming units; USAMRIID, United States Army Medical Research Institute for Infectious Disease; GP, glycoprotein; NHP, non-human primate.

All animals received two immunizations with an 8-week interval. The negative control group received an Ad26 empty vector followed by an MVA empty vector. The other three groups received either the Ad26.Filo, MVA-BN-Filo; Ad26.Filo, Ad35.Filo; or the MVA-BN-Filo, Ad26.Filo combination. Adenovirus vaccines were given at 1.2 × 10^11^ vp (4 × 10^10^ vp/vector) and the MVA-BN-Filo vector at a dose of 5 × 10^8^ infU. Four weeks after the second dose, the animals were challenged i.m. with 1000 pfu SUDV Gulu. Within 10 days after infection, all animals in the negative control group succumbed to infection (Figure 1A). Animals receiving the Ad26.Filo, MVA-BN-Filo or Ad26.Filo, Ad35.Filo regimen all survived the lethal challenge (100% protection), while the reverse order of vaccination (MVA-BN-Filo, Ad26.Filo) was less protective (three of five animals survived, 60% protection). The seven non-survivors (the five negative control animals and two animals of the group vaccinated with MVA-BN-Filo, Ad26.Filo) started to show clinical symptoms 6 or 7 days after challenge. Of all animals that were fully protected against SUDV challenge, one animal of the Ad26.Filo, Ad35.Filo group showed transient mild symptoms (clinical score 1 of 4, scoring system of USAMRIID) from days 7 to 10 (Figure 1B).

To assess immunogenicity, GP-specific binding antibody concentrations against SUDV Gulu were measured by ELISA in serum of all animals at five different time points: before immunization, 4 and 8 weeks after dose 1, and 2 and 3 weeks after dose 2 (Figure 1C). All three vaccine regimens tested (Ad26.Filo, MVA-BN-Filo; MVA-BN-Filo, Ad26.Filo; and Ad26.Filo, Ad35.Filo) showed high levels of SUDV GP–specific antibodies at 3 weeks post-dose 2, which is study week 11, 1 week prior to challenge (ranging from 3.31 to 4.14 log10 ELISA units [EU]/mL). Also 3 weeks post-dose 2, all vaccine regimens elicited SUDV-neutralizing antibodies (Supplementary Figure S1A). The group that received the MVA-BN-Filo, Ad26.Filo regimen showed low levels of SUDV GP–specific binding antibodies 8 weeks after dose 1 (ranging from 1.57 to 2.51 compared to a range of 2.24 to 2.85 log10 EU/mL for the other two groups) (Figure 1C). GP-specific binding antibody responses against the other filoviruses were analyzed as well. Similar patterns were observed for the EBOV Mayinga and MARV GP–specific antibody concentrations, with Ad26.Filo as dose 1 inducing higher antibody levels compared to MVA-BN-Filo as dose 1 for both EBOV and MARV (Supplementary Figure S1B,C). EBOV and MARV GP–specific antibody levels 3 weeks post-dose 2 (study week 11, 1 week prior to challenge) were very similar between the three different vaccine regimens, with only the Ad26.Filo and Ad35.Filo regimen trending somewhat higher for MARV GP–specific antibodies.

### 3.2. Immunogenicity and Protective Efficacy against MARV Challenge

Next, we investigated whether the Ad26.Filo, MVA-BN-Filo 0, 56 day vaccine regimen also provided full protection against MARV infection. For that purpose, 16 cynomolgus macaques were assigned to four groups: one control group (n = 2) and three groups each receiving a different vaccine regimen (Table 2). The negative control group received an Ad26 empty vector followed by Tris buffer as dose 2.

**Figure 2:**
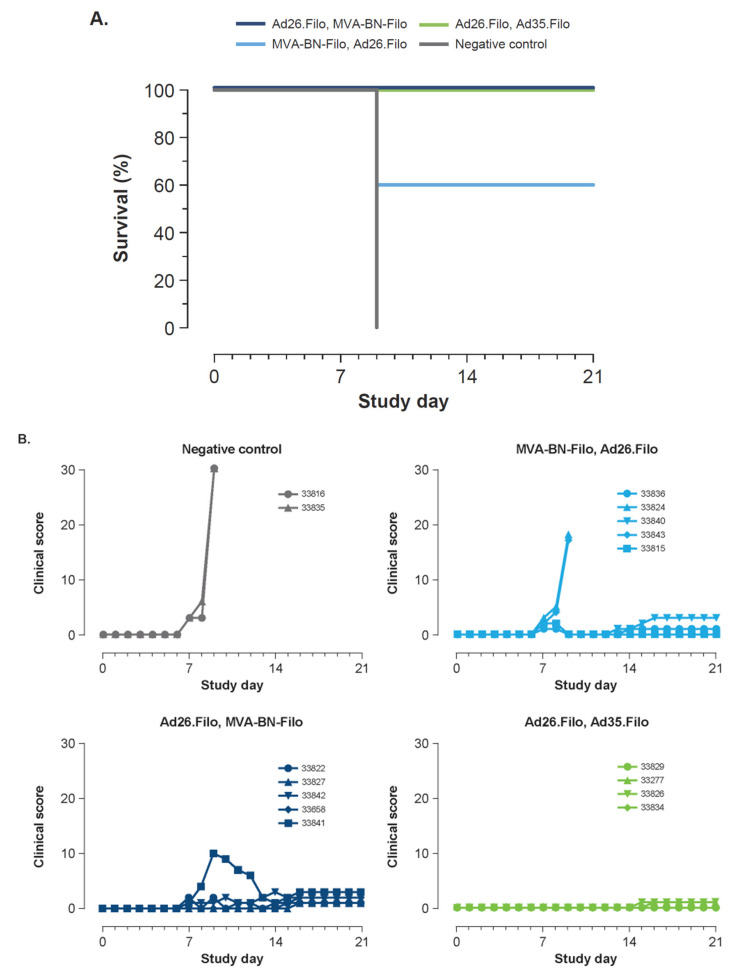

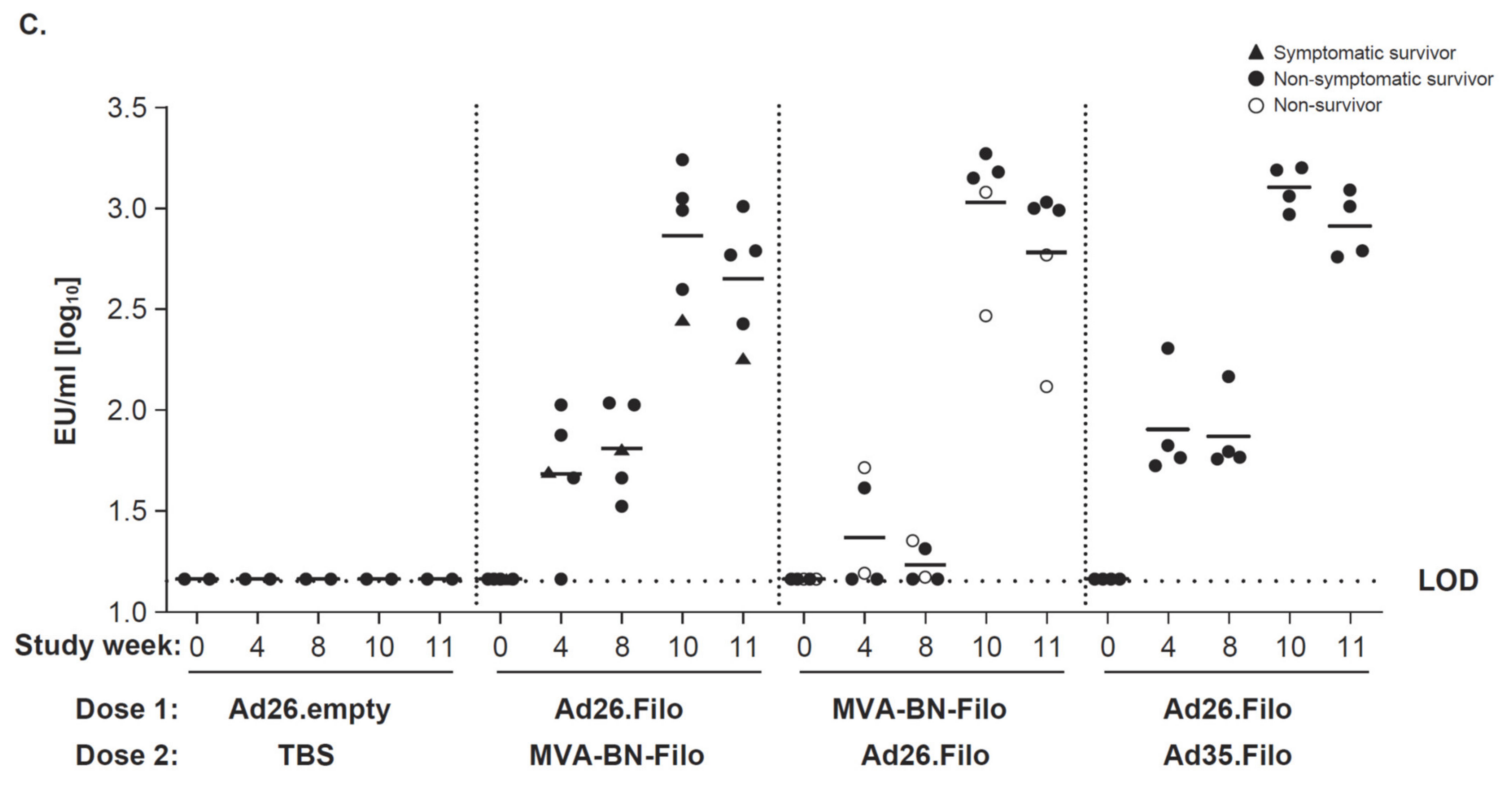
A trivalent two-dose heterologous regimen fully protects against MARV challenge. (**A**–**C**) Fourteen cynomolgus macaques were immunized i.m. with a two-dose trivalent vaccine combination of Ad26.Filo, MVA-BN-Filo (n = 5); MVA-BN-Filo, Ad26.Filo (n = 5); or Ad26.Filo, Ad35.Filo (n = 4) with an 8-week interval. Two cynomolgus macaques were included as negative controls and received Ad26 empty vector at week 0 and TBS at week 8. Adenovirus vaccines were given at 1.2 × 10^11^ vp (4 × 10^10^ vp/vector) and the MVA-BN-Filo vector at a dose of 5 × 10^8^ infU. A challenge with 1000 pfu MARV Angola was given i.m. 4 weeks post-dose 2. (**A**) Kaplan-Meier representation of survival. (**B**) Clinical scoring of individual animals after lethal challenge. (**C**) Humoral immune response over time measured by MARV GP–specific ELISA. Each symbol represents an individual NHP at the indicated time point, with the closed circles representing the non-symptomatic survivors, open circles the non-survivors, and triangles the symptomatic survivors (symptomatic is defined as clinical score >0). The black horizontal line indicates means per group, and the dashed horizontal line represents the LOD. Ad26, adenoviral vector serotype 26; MVA, modified vaccinia Ankara; Ad35, adenoviral vector serotype 35; EU, enzyme-linked immunosorbent assay unit; LOD, limit of detection; MARV, Marburg virus; i.m., intramuscularly; TBS, Tris-buffered saline; vp, viral particle; infU, infectious unit; pfu, plaque-forming units; GP, glycoprotein; ELISA, enzyme-linked immunosorbent assay; NHP, non-human primate.

The three groups immunized with vaccine regimens received either Ad26.Filo, Ad35.Filo (n = 4); Ad26.Filo, MVA-BN-Filo (n = 5); or MVA-BN-Filo, Ad26.Filo regimen (n = 5). Adenovirus vaccines were given at 1.2 × 10^11^ vp (4 × 10^10^ vp/vector) and the MVA-BN-Filo vector at a dose of 5 × 10^8^ infU. Four weeks after dose 2, the animals were challenged i.m. with 1000 pfu MARV Angola. On day 9 post-challenge, both negative control animals succumbed to infection. Additionally, at day 9 post-challenge, two of the five animals of the MVA-BN-Filo, Ad26.Filo group succumbed to infection (Figure 2A). The remaining 12 vaccinated animals survived until the end of the study. Minimal morbidity was observed in seven of the eight survivors (clinical score up to 3, scoring system at TBRI), whereas 1 NHP had substantial morbidity (clinical score up to 10; Figure 2B). Additionally, the efficacy of a single dose of Ad26 was suggested by a study in which a single administration of an Ad26 vector encoding the MARV GP was tested. NHP were immunized with Ad26.MARVA (n = 4, dose of 1 × 10^11^ vp), Ad5.MARVA (n = 1, dose of 1 × 10^11^ vp), or diluent buffer (n = 1) and challenged 5 weeks post-immunization with 1000 pfu MARV Angola (Supplementary Figure S2). The negative control animal succumbed to infection 10 days after infection, and the five immunized animals survived.

Immunogenicity of the two-dose immunization regimens prior to infection was investigated by analyzing both the humoral and cellular immune responses (Figure 2C and Figure S3). The MARV GP–specific antibody levels induced by the three different heterologous vaccine regimens were very similar 3 weeks post-dose 2 (study week 11; Figure 2C). However, the MARV GP–specific antibody levels induced post-dose 1, at study weeks 4 and 8, were lower post-MVA compared to post-Ad26 (Figure 2C). One animal of the Ad26.Filo, MVA-BN-Filo regimen group (NHP 33841), that experienced transient severe clinical symptoms from day 8 until day 12 after infection, showed the lowest MARV GP–specific antibody concentration in serum at weeks 10 and 11 from the 5 animals in that group (2.45 and 2.26 EU/mL [log10]). Additionally, MARV-specific neutralizing antibodies could be detected in all immunized animals except for two animals in the MVA-BN-Filo, Ad26.Filo group (Supplementary Figure S3A).

For EBOV and SUDV GP–specific antibody levels, similar patterns to MARV GP–specific antibody levels were observed (Supplementary Figure S3B,C), with a reduced GP-specific antibody level post-MVA dose 1 for EBOV GP–specific antibodies and, to a lesser extent, for SUDV GP–specific antibodies. No clear differences were detected in induced antibody levels between the three vaccine regimens 3 weeks post-dose 2 (study week 11) for both EBOV and SUDV (Supplementary Figure S3B,C). In addition to the humoral response, all three vaccination regimens induced specific cellular responses against EBOV, SUDV, and MARV (Supplementary Figure S3D). At 3 weeks post-dose 2 (week 11, 1 week prior to challenge), antigen-specific IFN-γ T-cell responses were detected for EBOV, SUDV, and MARV in all the vaccinated animals included in the study, with a large variation between individual animals.

### 3.3. Marburg and Sudan GP–Specific Antibody Responses Correlate with Protection

**Figure 3:**
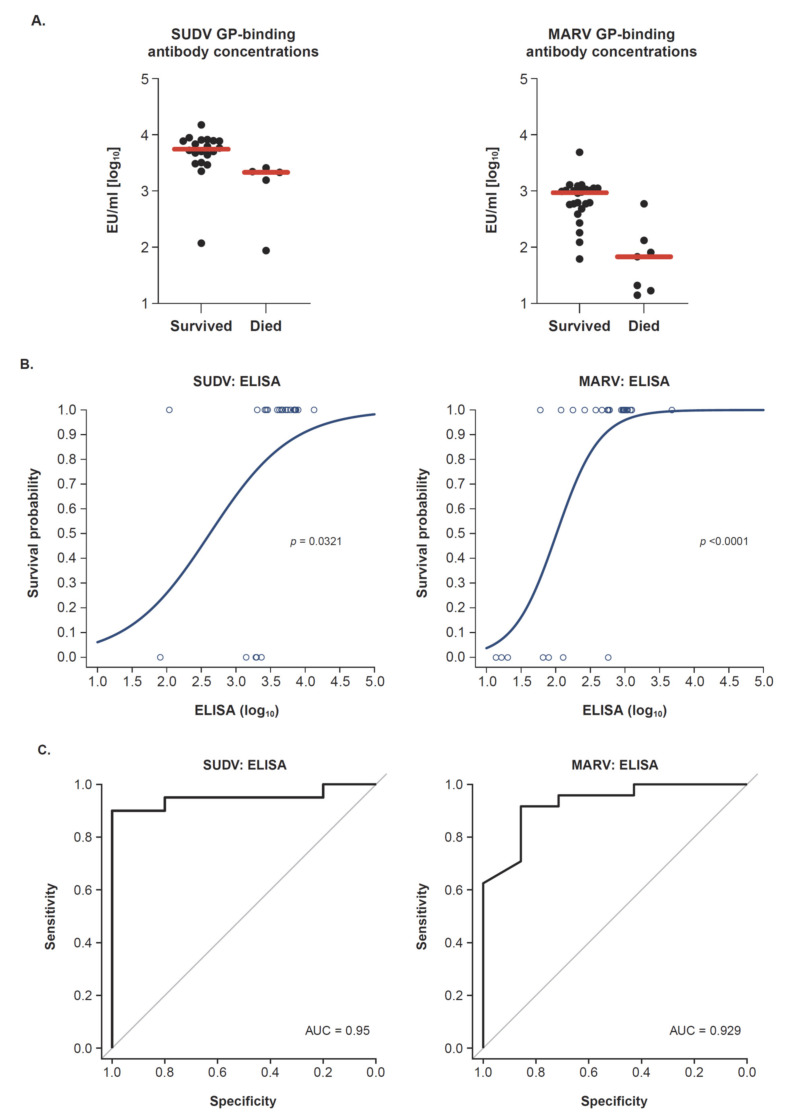
Immunological correlates of protection in NHP. (**A**) MARV and SUDV GP–binding antibody concentrations (EU/mL, log10 transformed) measured at 1 week prior to challenge are indicated for surviving and non-surviving animals. Each dot represents an animal, and the horizontal red line indicates the median of the group. Data are obtained from five independent studies including different two-dose trivalent heterologous vaccine regimens. (**B**) Graphs represent logistic regression models with SUDV GP–specific binding antibody concentrations and MARV GP–specific binding antibody concentrations as predictors of survival after SUDV challenge (1000 pfu, i.m.) and MARV challenge (1000 pfu, i.m.) in cynomolgus macaques. The *p*-values refer to the slopes of the logistic regression models. (**C**) AUC of the ROC curves for sensitivity and specificity of GP-binding antibody levels in predicting survival after challenge. SUDV, Sudan virus; GP, glycoprotein; MARV, Marburg virus; EU, ELISA unit; ELISA, enzyme-linked immunosorbent assay; AUC, area under the curve; NHP, non-human primate; pfu, plaque-forming units; i.m., intramuscular; ROC, receiver operating characteristic.

We have previously demonstrated that the EBOV GP–specific antibody concentration after a heterologous two-dose vaccine regimen (Ad26.ZEBOV, MVA-BN-Filo) was a strong predictor of survival after challenge of cynomolgus macaques with EBOV [14]. To examine whether similar predictions may apply to the SUDV and MARV-specific challenges, data from five independent studies (two SUDV and three MARV studies as represented in this article and as previously published [13]) were analyzed for a correlation between GP-binding antibody concentrations and survival. A summary of vaccine regimens for all NHP included in the analysis is provided in Supplementary Tables S1 and S2. Animals immunized with control adenovectors (empty vectors) were excluded from analysis. While the time between vaccination and challenge was different between experiments, it was assumed that the pre-challenge immune response levels had direct relevance for protection. GP-binding antibody concentrations measured 1 week prior to the challenge of animals immunized with a two-dose heterologous regimen consisting of combinations of Ad26.Filo, Ad35.Filo, and/or MVA-BN-Filo were included in the analysis. Positive control animals receiving a single administration of monovalent Ad5 encoding SUDV or MARV were also included. The median GP-specific antibody concentration of non-survivors was lower than that of survivors in both the SUDV and MARV experiments (Figure 3A). To further explore the relationship between the concentration of GP-binding antibodies and survival, a logistic regression analysis was performed. This analysis describes the probability of survival as a function of vaccine immunogenicity as measured by GP-binding antibody concentration. Survival was used as a binary outcome (0 = death; 1 = survival) with the GP-binding concentration as a covariate. A significant positive slope for the logistic model indicates that increasing levels of immunogenicity are positively correlated with survival. GP-binding antibody levels significantly correlated with the challenge outcome for SUDV (n = 25; slope, *p* = 0.0321) and also for MARV (n = 31; slope, *p* < 0.0001; Figure 3B). The correlation between GP-binding antibody concentrations and survival was further corroborated by good sensitivity and specificity as shown by ROC AUC values of 0.95 and 0.929 for SUDV and MARV, respectively (Figure 3C).

In conclusion, the SUDV and MARV GP–specific binding antibody concentrations are good predictors for survival outcome after challenge, even when analyzed across various vaccination regimens.

### 3.4. Protective Vaccine Regimens Induce Persistent Immune Responses

For the two-dose heterologous vaccine regimen (Ad26.Filo, MVA-BN-Filo), which was shown to be fully protective against EBOV, SUDV, and MARV challenge [13] (Figure 1 and Figure 2), immunogenicity against EBOV GP, MARV GP, and SUDV GP was followed over time. Cynomolgus macaques were immunized with the trivalent two-dose heterologous vaccine regimen (Ad26.Filo, MVA-BN-Filo). Additional groups (n = 5) received a monovalent Ad26 dose 1 (Ad26.SUDV, Ad26.MARV, or Ad26.ZEBOV) followed by the MVA-BN-Filo as dose 2 with an 8-week interval. Immunogenicity to the relevant homologous strain was followed longer, while immunogenicity for the non-homologous strains was measured at two time points (4 weeks post-dose 1 and 3 weeks post-dose 2). Finally, five unvaccinated negative control animals were included (saline, n = 5; Table 3).

**Figure 4:**
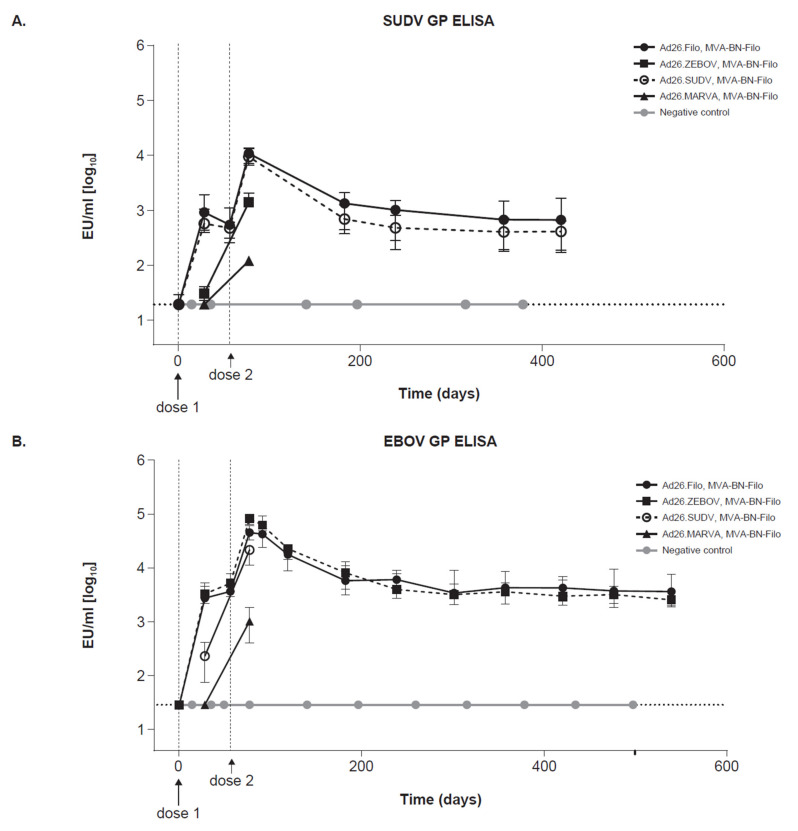

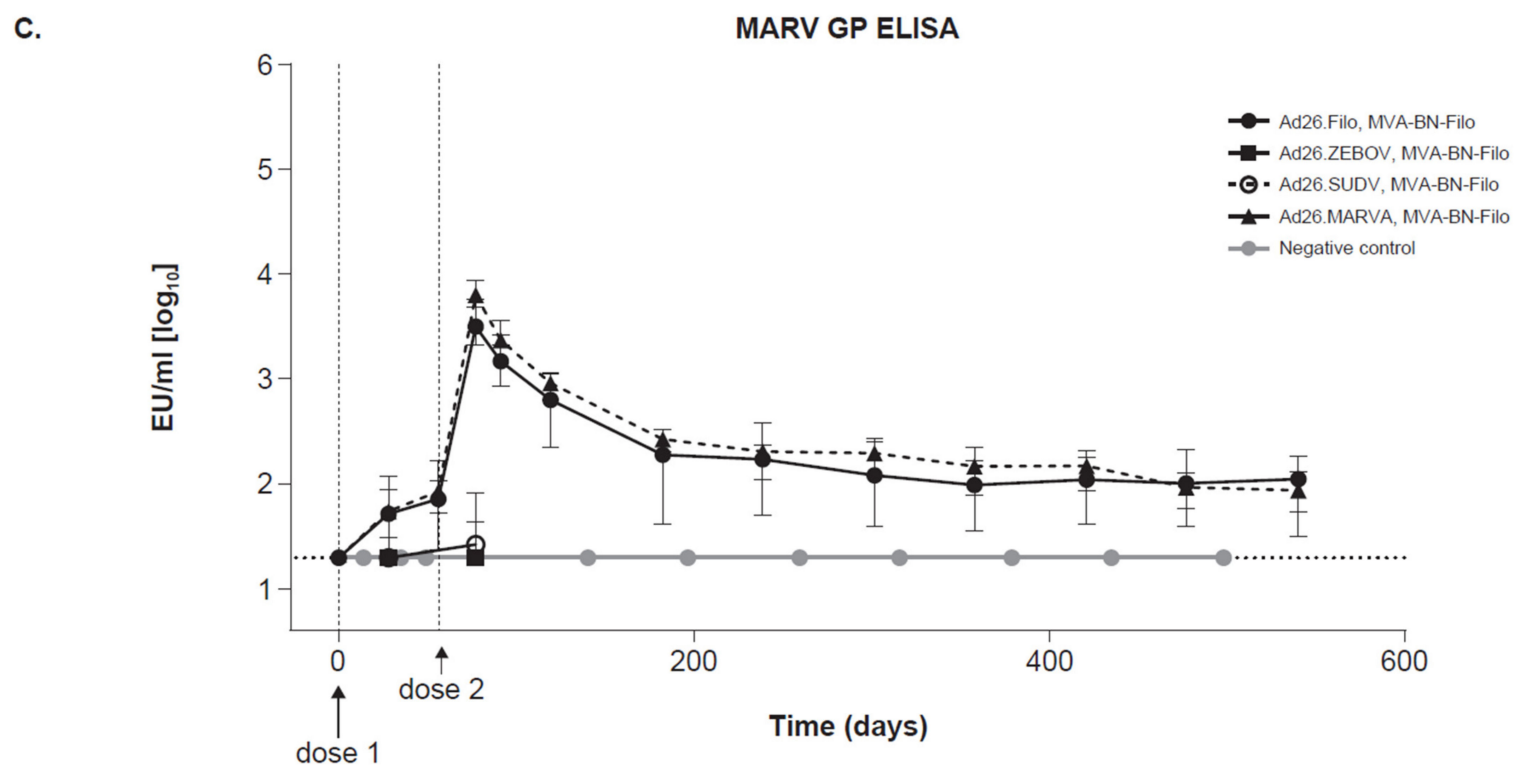
Durable immunogenicity. GP-binding antibodies specific to (**A**) SUDV, (**B**) EBOV, and (**C**) MARV. Cynomolgus macaques were immunized with a two-dose vaccine regimen with an 8-week interval between dose 1 and dose 2, and GP-specific binding antibody responses were monitored up to day 421 (SUDV) or day 540 (EBOV and MARV). One group (n = 10) received the trivalent Ad26.Filo (1.2 × 10^11^ vp) followed by MVA-BN-Filo (5 × 10^8^ infU) as dose 2. Three other groups received a monovalent Ad26 (Ad26.ZEBOV, Ad26.SUDV, or Ad26.MARVA) at 4 × 10^10^ vp followed by MVA-BN-Filo (5 × 10^8^ infU) (n = 5 per group). A negative control group (n = 5) received two injections with saline, 2 weeks apart. GP-specific binding antibodies are measured by ELISA and median and interquartile range are indicated per group over time. SUDV, Sudan virus; GP, glycoprotein; ELISA, enzyme-linked immunosorbent assay; EU, ELISA unit; Ad26, adenoviral vector serotype 26; MVA, modified vaccinia Ankara; EBOV, Ebola virus; MARV, Marburg virus; vp, viral particle; infU, infectious units.

GP-binding antibodies (Figure 4A–C) and neutralizing antibodies (Supplementary Figure S4A–C) to SUDV, EBOV, and MARV were measured from the study start (pre-immunization) until day 421 for SUDV and day 540 for EBOV and MARV. For SUDV GP–specific binding antibodies, both the trivalent and the SUDV monovalent dose 1 regimen induced similarly high antibody levels at 3 weeks post-dose 2 (Figure 4A). Monovalent EBOV and MARV dose 1 regimens also induced SUDV GP–specific binding antibodies 3 weeks post-dose 2, albeit to a lower extent than the trivalent and SUDV monovalent dose 1 regimens. Results from the long-term follow-up time points showed that the level of SUDV GP–specific antibodies remained stable until the end of the study (day 421).

For EBOV GP–specific binding antibodies, the trivalent and monovalent (EBOV and SUDV) dose 1 regimens induced similarly high antibody levels at 3 weeks post-dose 2 (Figure 4B). The monovalent Ad26.MARVA dose 1 regimen did induce EBOV GP–specific binding antibodies at 3 weeks post-dose 2, but to a lower level compared to the other three regimens at that time point (Figure 4B). The similar response with the Ad26.ZEBOV and Ad26.SUDV monovalent dose 1 regimen could be partially attributed to the high homology between the EBOV and SUDV GP DNA sequences. The EBOV GP–binding antibody concentrations induced by the trivalent and monovalent Ad26.ZEBOV dose 1 regimen remained stable until day 521.

MARV GP–specific binding antibodies were induced by the trivalent as well as the monovalent Ad26.MARVA dose 1 regimens, with the highest detectable levels at 3 weeks post-dose 2 (Figure 4C). The monovalent (Ad26.SUDV and Ad26.ZEBOV) dose 1 regimens did not induce a MARV GP–specific binding antibody response at 3 weeks post-dose 2. MARV GP–specific binding antibody levels induced by the trivalent and monovalent Ad26.MARVA dose 1 regimen remained stable until the end of the study (day 521).

Neutralizing GP-specific antibodies against SUDV, EBOV, and MARV were induced by the trivalent and monovalent dose 1 regimens (specific for the homologous strain) (Supplementary Figure S4A–C). Like the pattern observed for the GP-specific binding antibodies, the highest antibody levels were measured at 3 weeks post-dose 2, and levels remained stable until the end of the study for SUDV and EBOV. For the neutralizing MARV GP–specific antibody levels, the highest antibody levels were also measured at 3 weeks post-dose 2, yet the antibody levels decreased post-day 200 and did not remain at a stable plateau as was observed for the GP-binding antibodies.

Overall, the heterologous trivalent two-dose regimen (Ad26.Filo, MVA-BN-Filo) induced durable GP-specific binding antibody concentrations against SUDV, EBOV, and MARV for at least 421 (SUDV) and 540 days (EBOV and MARV) after immunization.

## 4. Discussion

Here we provide evidence that a two-dose heterologous trivalent vaccine regimen (Ad26.Filo, MVA-BN-Filo) induces a protective immune response in NHP against both SUDV and MARV in addition to the already reported protection against EBOV [13]. This vaccine regimen induced high humoral immune responses, as characterized by high levels of GP-specific binding antibodies and GP-specific neutralizing antibodies 3 weeks post-dose 2. The vaccine-induced GP-binding antibody levels were good predictors of challenge outcome as indicated by the correlation between antibody levels and survival outcome and by the high discriminatory capacity of the logistic model (Figure 3B,C). Moreover, the elicited GP-specific binding antibody response remained stable for more than 1 year for all three filovirus GPs included in the regimen, EBOV, SUDV, and MARV.

A vaccine-induced cellular immune response, as determined by antigen-specific IFN-γ T-cell responses, was elicited by the heterologous two-dose Ad26.Filo, MVA-BN-Filo regimen for the three viruses (EBOV, SUDV, and MARV) in all animals 1 week prior to challenge (Supplementary Figure S3D). As the cynomolgus macaques are an outbred population, the magnitude of the induced cellular responses to the various filovirus-specific GPs were highly heterogeneous between individual animals.

Ad26.Filo as a dose 1 induced a strong immune response, and a monovalent 1-dose vaccination with Ad26.MARVA protected against MARV infection when administered at a high dosage (Supplementary Figure S2). Other monovalent vaccines were also reported to provide full protection in NHP against lethal challenge with MARV [24,25,26] and EBOV [27]. Single-dose vaccine regimens have clear advantages if used for reactive vaccination in an outbreak situation. While a single administration was protective to MARV [26], differences between the vectors were reflected in the different dosages needed to achieve full protection [27]. Complementing a single-dose vaccine regimen with a second heterologous dose clearly enhanced the immune response (as demonstrated by the high GP-specific binding antibody concentration at 3 weeks post-dose 2; Figure 4). The addition of a second (heterologous) vaccine administration can lower the required vaccine dosage and increase the level of protection as measured by GP-specific binding antibodies [14]. Therefore, in a prophylactic setting, lower doses can be used and full protection provided by two-dose regimens.

While the mechanistic correlates of protection against filovirus disease have not been fully characterized and may differ between vaccines, GP-binding antibodies have emerged as a good immunological marker for protection against EBOV [14] and MARV [26]. Although other immunological markers, such as the neutralizing antibody concentration and cellular response (IFN-γ T-cell response) also have predictive capacities, the GP-binding antibody concentration is documented as a robust immune correlate of protection with a high discriminatory capacity. The importance of an adequate antibody response at the time of infection is further emphasized in this MARV challenge study, in which one of the surviving NHP of the Ad26, MVA vaccinated group (Animal ID 33841) transiently showed clinical symptoms after challenge (Figure 2B). This NHP mounted a detectable cellular response (as measured by ELISpot) but showed the lowest antibody response in that group (2.26 EU/mL [log10] at week 11; Figure 2C). Although this NHP did survive the MARV challenge, which was lethal for untreated animals, and virus could not be detected in serum at any point during the study, the occurrence of clinical symptoms suggests that a low antibody response may be a risk factor for a fatal outcome of viral disease in NHP. The correlation between GP-specific binding antibodies for SUDV and MARV and prediction of survival outcome in the studies described here, together with the high discriminatory capacity (as evidenced by a high AUC of 0.95 for SUDV and 0.93 for MARV), indicates that binding antibody concentrations could be a suitable marker for protection. This would require additional confirmation, as the number of observations is relatively low. In addition, the overlap in ranges of antibody concentrations between survivors and non-survivors would likely preclude setting a threshold for protection.

The highest detected level of the GP-binding antibody concentration for SUDV, EBOV, and MARV was observed at 2 to 3 weeks post-dose 2, followed by a decline. Stable antibody concentrations were detected from day 184 and lasted at least for more than 1 year. Interestingly, GP-specific antibody levels as induced by the trivalent vaccine regimen (Ad26.Filo, MVA-BN-Filo) were comparable to the monovalent dose 1 regimens (Ad26.SUDV; Ad26.ZEBOV; or Ad26.MARVA, MVA-BN-Filo) for all three virus GPs (SUDV, EBOV, and MARV). There was no apparent difference in the kinetics of the GP-specific binding antibodies between the regimens of monovalent or trivalent vaccination. For SUDV and EBOV, a monovalent vaccination induced GP-specific antibodies to the specificity not incorporated in the Ad26 vaccine vector. Particularly, the Ad26.ZEBOV, MVA-BN-Filo regimen induced high levels of SUDV GP-specific antibodies, and the Ad26.SUDV, MVA-BN-Filo regimen resulted in high levels of EBOV GP–specific antibodies. These responses are most likely due to homology of the GP regions of the SUDV and EBOV used in the adenoviral vectors, as well as boosting with the heterologous vector. However, the observed immunological cross-reactivity may not automatically translate into cross-protection, as shown previously using a single administration of live-attenuated EBOV vaccine, which was fully protective for EBOV Kikwit but failed to induce protection against SUDV [28].

Overall, a regimen consisting of a monovalent dose 1 and a trivalent dose 2 has similar GP-specific binding antibody levels as a two-dose trivalent vaccine regimen against the antigen included in the monovalent dose 1. However, the benefit of inducing high immunogenicity towards all three filoviruses is lost when using a monovalent dose 1; this is especially evident for Ad26.MARVA. Moreover, the possibility of inducing sufficient levels of protection against multiple filoviruses may be lost when using monovalent vaccines instead of trivalent vaccines.

A first generation prime-boost, gene-based, multivalent filovirus vaccine included EBOV, SUDV, and Tai Forest virus GPs, and uniformly protected macaques from lethal EBOV challenge [29]. Subsequent early efforts towards a multivalent vaccine have been conducted with an Ad5-based adenoviral construct. At first, a bivalent Ad5-based vector, encoding the GP sequences of EBOV and SUDV, was demonstrated to be immunogenic for both EBOV and SUDV and efficacious against EBOV challenge in mice [30]. Two years later, an Ad5-based “pan-filovirus” vaccine formulation was generated, consisting of four vectors that cumulatively express five filovirus GP antigens (EBOV, SUDV, and three MARV strains: Ci67 and Musoke, and RAVV) and two filovirus NP antigens (EBOV Kikwit and MARV Musoke). Immunized cynomolgus macaques survived a MARV Musoke or EBOV Kikwit challenge 15 weeks after the two-dose homologous vaccine regimen. This study further strengthens the finding that immunization with a multivalent vaccine can efficiently protect NHP against lethal filovirus challenge. Interestingly, vaccine-induced GP-binding antibody levels reached a plateau before the administration of the second dose and remained high until the end of the study (day 203) [31]. In our hands, a two-dose heterologous regimen increased GP-specific binding antibody levels. Despite these promising NHP data with the Ad5-based vector, due to the high pre-existing immunity in humans against Ad5 worldwide, a vaccine using other adenoviral vector serotypes, such as Ad26 or ChAd3 [26], may be the preferred choice for clinical development.

## 5. Conclusions

We show here that vaccination with a two-dose regimen confers long-lasting immune responses against three different species of filovirus in NHP. A phase I trial that assessed safety, tolerability, and immunogenicity of the Ad26.Filo, MVA-BN-Filo regimen vaccine (ClinicalTrials.gov (accessed on 29 June 2022) Identifier: NCT02860650) was recently completed. This study showed that the Ad26.Filo, MVA-BN-Filo regimen was safe and well tolerated by all participants. Furthermore, 21 days post-dose 2, 100% of participants on the active regimen responded to vaccination and exhibited binding antibodies against EBOV, SUDV, and MARV GPs [17]. In combination, both the NHP and the human data indicate that the Ad26.Filo, MVA-BN-Filo regimen may be a multivalent prophylactic vaccination strategy in regions at high risk of filovirus outbreaks.

## Figures and Tables

**Table 1:** Study design of SUDV challenge.

Group	N	Day 0 Immunization	Day 56 Immunization	Challenge
A	5	Ad26.empty	MVA.empty	SUDV Gulu *
B	5	Ad26.Filo	MVA-BN-Filo	SUDV Gulu *
C	5	MVA-BN-Filo	Ad26.Filo	SUDV Gulu *
D	5	Ad26.Filo	Ad35.Filo	SUDV Gulu *

* SUDV Gulu challenge on day 84 at a dose of 1000 pfu. SUDV, Sudan virus; Ad26, adenoviral vector serotype 26; Ad35, adenoviral vector serotype 35; MVA, modified vaccinia Ankara; pfu, plaque-forming unit.

**Table 2:** Study design of MARV challenge.

Group	N	Day 0 Immunization	Day 56 Immunization	Challenge
A	2	Ad26.empty	TBS	MARV Angola *
B	5	Ad26.Filo	MVA-BN-Filo	MARV Angola *
C	5	MVA-BN-Filo	Ad26.Filo	MARV Angola *
D	4	Ad26.Filo	Ad35.Filo	MARV Angola *

* MARV Angola challenge on day 84 at a dose of 1000 pfu. MARV, Marburg virus; Ad26, adenoviral vector serotype 26; MVA, modified vaccinia Ankara; Ad35, adenoviral vector serotype 35; TBS, Tris-buffered saline; pfu, plaque-forming unit.

**Table 3:** Study design of long term immunogenicity.

Group	N	Day 0 Immunization	Day 56 Immunization
1	10	Ad26.Filo	MVA-BN-Filo
2	5	Ad26.ZEBOV	MVA-BN-Filo
3	5	Ad26.SUDV	MVA-BN-Filo
4	5	Ad26.MARVA	MVA-BN-Filo
5	5	Saline	Saline

Ad26, adenoviral vector serotype 26; MVA, modified vaccinia Ankara.

## Data Availability

All relevant data are within the paper and its Supplementary Materials files.

## Supplementary Materials

The following supporting information can be downloaded at: https://www.mdpi.com/article/10.3390/vaccines10081263/s1, Table S1: SUDV GP–binding antibody concentrations measured 1 week prior to challenge; Table S2: MARV GP–binding antibody concentrations measured 1 week prior to challenge; Figure S1: SUDV-neutralizing antibodies and EBOV Mayinga and MARV GP–specific antibody concentrations; Figure S2: Efficacy of a single administration of an Ad26 vector encoding the MARV GP; Figure S3: Humoral and cellular immune responses; Figure S4: Neutralizing GP-specific antibodies against SUDV, EBOV, and MARV.
