# Supplementary material for: Protection against Marburg Virus and Sudan Virus in NHP by an Adenovector-Based Trivalent Vaccine Regimen Is Correlated to Humoral Immune Response Levels
Source: Vaccines (Basel). 2022 Aug 5;10(8):1263. doi: 10.3390/vaccines10081263 (PMC9412258; doi:10.3390/vaccines10081263)
Supplement: Supplementary file 1 [file vaccines-10-01263-s001.zip › vaccines-1820237-supplementary.pdf]

Supplementary Table S1. SUDV GP–binding antibody concentrations measured 1 week prior to challenge.

SUDV Study	Animal ID	Dose 1	Dose 2	Immunization Schedule (days)	SUDV GP–Binding Concentration EU/mL [log 10] 1 Week Prior to Challenge	Survival (Y/N)	Reference
**8**	32236	Ad26.Filo	Ad35.Filo	0, 28	3.85	Y	Callendret et al. 2018 [1]
	32239	Ad26.Filo	Ad35.Filo	0, 28	3.91	Y	
	32240	Ad26.Filo	Ad35.Filo	0, 28	3.73	Y	
	32242	Ad26.Filo	Ad35.Filo	0, 28	3.38	N	
	32235	Ad26.Filo	Ad26.Filo	0, 28	3.67	Y	
	32237	Ad26.Filo	Ad26.Filo	0, 28	3.47	Y	
	32241	Ad26.Filo	Ad26.Filo	0, 28	3.16	N	
	32234	Ad26.Filo	Ad26.Filo	0, 28	3.32	Y	
	32300	none	Ad5.SEBOV	0, 28	1.92	N	
	32244	none	Ad5.SEBOV	0, 28	2.05	Y	
**29#4**	AL403C	Ad26.Filo	MVA-BN-Filo	0, 56	3.86	Y	Figure 1
	AB9851	Ad26.Filo	MVA-BN-Filo	0, 56	3.76	Y	
	AK990H	Ad26.Filo	MVA-BN-Filo	0, 56	4.14	Y	
	AL765D	Ad26.Filo	MVA-BN-Filo	0, 56	3.80	Y	
	BF944C	Ad26.Filo	MVA-BN-Filo	0, 56	3.85	Y	
	BF902C	MVA-BN-Filo	Ad26.Filo	0, 56	3.31	N	
	AS96B	MVA-BN-Filo	Ad26.Filo	0, 56	3.67	Y	
	AP128C	MVA-BN-Filo	Ad26.Filo	0, 56	3.45	Y	
	AJ786F	MVA-BN-Filo	Ad26.Filo	0, 56	3.30	N	
	AL789F	MVA-BN-Filo	Ad26.Filo	0, 56	3.88	Y	
	BF855B	Ad26.Filo	Ad35.Filo	0, 56	3.43	Y	
	AB786M	Ad26.Filo	Ad35.Filo	0, 56	3.61	Y	
	BF842C	Ad26.Filo	Ad35.Filo	0, 56	3.69	Y	
	C912HF	Ad26.Filo	Ad35.Filo	0, 56	3.87	Y	
	AC21K	Ad26.Filo	Ad35.Filo	0, 56	3.64	Y	

SUDV, Sudan virus; GP, glycoprotein; EU, enzyme-linked immunosorbent assay unit; Y, yes; N, no; Ad26, adenoviral vector serotype 26; Ad35, adenoviral vector serotype 35; Ad5 adenoviral vector serotype 5, MVA, modified vaccinia Ankara.

Supplementary Table S2. MARV GP–binding antibody concentrations measured 1 week prior to challenge.

MARV Study	Animal ID	Dose 1	Dose 2	Dose 3	Immunization Schedule (days)	MARV GP-Binding Concentration EU/mL [log10] 1 Week Prior to Challenge	Survival (Y/N)	Reference
**6A**	31611	Ad26 tetravalent	Ad35 tetravalent	-	0, 28*	1.79	Y	Callendret et al. 2018 [1]
	31612	Ad26 tetravalent	Ad35 tetravalent	-	0, 28*	1.15	N	
	31614	Ad26 tetravalent	Ad35 tetravalent	-	0, 28*	1.32	N	
	31616	Ad26 tetravalent	Ad35 tetravalent	-	0, 28*	1.83	N	
	31617	Ad26 tetravalent	Ad35 tetravalent	-	0, 28*	1.91	N	
	31621	Ad26 tetravalent	Ad35 tetravalent	-	0, 28*	1.23	N	
	31870	Ad26.MARVA	Ad35.MARVA	-	0, 56	2.96	Y	
**6B**	AP34	Ad26 tetravalent	Ad35 tetravalent	Ad26 tetravalent	0, 28, C-4†	2.98	Y	Callendret et al. 2018 [1]
	AP528	Ad26 tetravalent	Ad35 tetravalent	Ad26 tetravalent	0, 28, C-4†	3.69	Y	
	AR120	Ad26 tetravalent	Ad35 tetravalent	Ad26 tetravalent	0, 28, C-4†	3.11	Y	
	AR968	Ad26 tetravalent	Ad35 tetravalent	Ad26 tetravalent	0, 28, C-4†	3.05	Y	
	AT709	Ad26 tetravalent	Ad35 tetravalent	Ad26 tetravalent	0, 28, C-4†	3.11	Y	
	BM515	Ad26 tetravalent	Ad35 tetravalent	Ad26 tetravalent	0, 28, C-4†	3.05	Y	
	A13960	-	-	Ad5.MARVA	C-4	2.09	Y	
	A13377	Ad26.Filo	Ad26.Filo	-	0, 28	2.59	Y	
	A13391	Ad26.Filo	Ad26.Filo	-	0, 28	2.77	Y	
	A12903	Ad26.Filo	Ad26.Filo	-	0, 28	2.68	Y	
**13**	33822	Ad26.Filo	MVA-BN-Filo	-	0, 56	2.79	Y	Figure 2
	33827	Ad26.Filo	MVA-BN-Filo	-	0, 56	3.01	Y	
	33842	Ad26.Filo	MVA-BN-Filo	-	0, 56	2.43	Y	
	33658	Ad26.Filo	MVA-BN-Filo	-	0, 56	2.77	Y	
	33841	Ad26.Filo	MVA-BN-Filo	-	0, 56	2.26	Y	
	33836	MVA-BN-Filo	Ad26.Filo	-	0, 56	3.03	Y	
	33824	MVA-BN-Filo	Ad26.Filo	-	0, 56	2.77	N	
	33840	MVA-BN-Filo	Ad26.Filo	-	0, 56	2.99	Y	
	33843	MVA-BN-Filo	Ad26.Filo	-	0, 56	2.12	N	
	33815	MVA-BN-Filo	Ad26.Filo	-	0, 56	3.00	Y	
	33829	Ad26.Filo	Ad35.Filo	-	0, 56	2.79	Y	

	33277	Ad26.Filo	Ad35.Filo	-	0, 56	3.09	Y	
	33826	Ad26.Filo	Ad35.Filo	-	0, 56	2.76	Y	
	33834	Ad26.Filo	Ad35.Filo	-	0, 56	3.01	Y	

*MARV challenge was performed 12 months after immunization.

†Animals received a tetravalent Ad26 booster 4 weeks before MARV challenge.

MARV, Marburg virus; GP, glycoprotein; EU, enzyme-linked immunosorbent assay unit; Y, yes; N, no; Ad26, adenoviral vector serotype 26; Ad35, adenoviral vector serotype 35; MVA, modified vaccinia Ankara.

Supplementary Figures

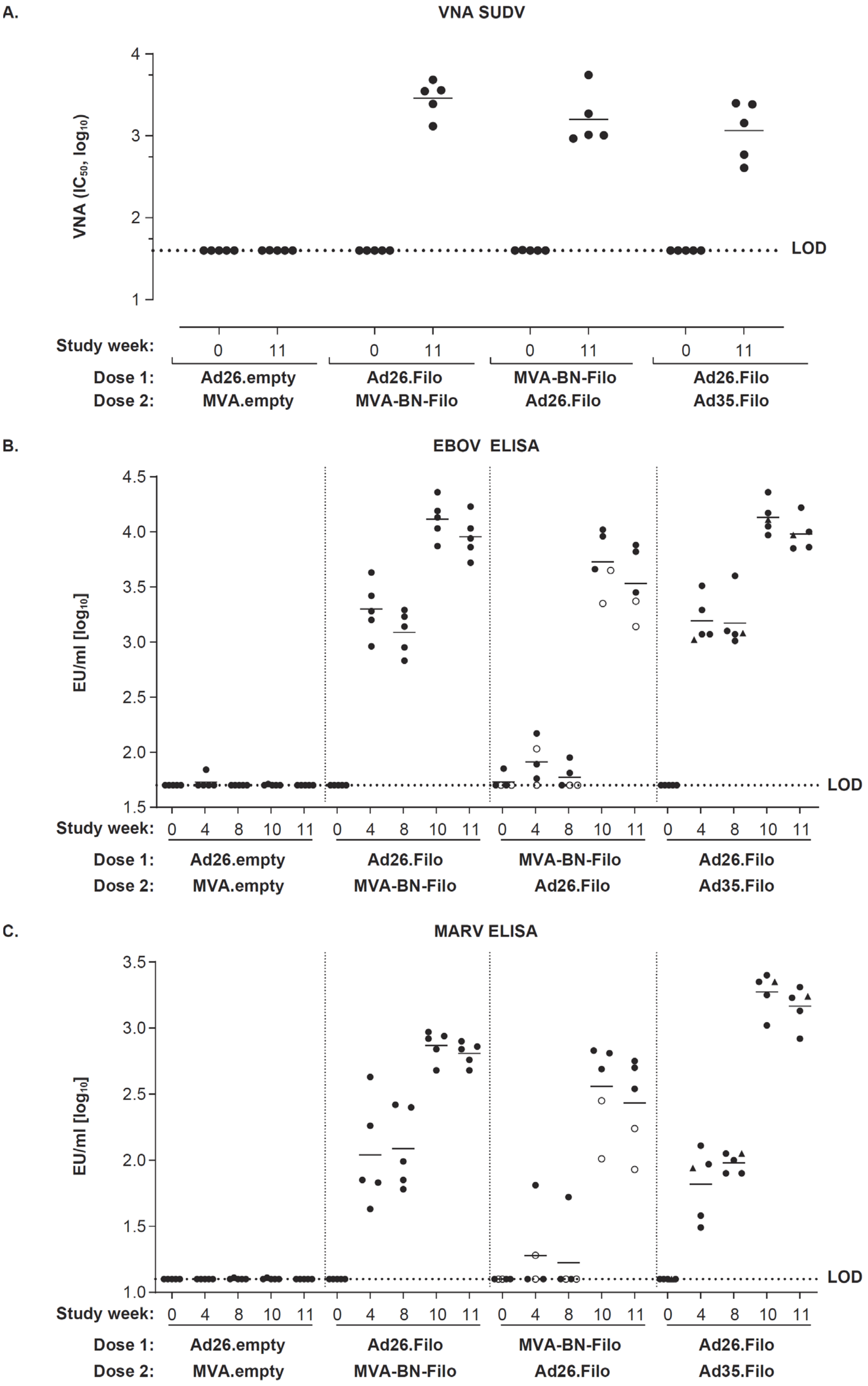

Supplementary Figure S1. SUDV-neutralizing antibodies and EBOV Mayinga and MARV GP–specific antibody concentrations. Twenty cynomolgus macaques were immunized i.m. with Ad26.Filo as dose 1 at week 0 and MVA-BN-Filo or Ad35.Filo as dose 2 at week 8, or MVA-BN-Filo as dose 1 and Ad26.Filo as dose 2, or empty Ad26 and empty MVA-BN vector (n = 5 per group). Adenovirus vaccines were given at 1.2×10^{11} vp (4×10^{10} vp/vector) and the MVA-

BN-Filo vector at a dose of 5 × 10^8 TCID50. (**A**) SUDV Gulu neutralization is expressed as IC_{50} titers on a log10 scale. The neutralization was measured in samples collected before dose 1 (t = 0) and 3 weeks post-dose 2 (week 11). The dotted line indicates lower limit of detection of the assay (LOD = 1.6 log10). Black bars indicate geometric mean of the titers within each group. (**B,C**) Humoral immune responses were measured over time by EBOV and MARV GP–specific ELISA. Each symbol represents an individual NHP at the indicated time point, with the closed circles representing the non-symptomatic survivors, open circles the non-survivors, and triangles the symptomatic survivors (symptomatic is defined as clinical score >0). Means per group are indicated with the black horizontal line, and the dashed horizontal line represents the LOD. VNA, virus neutralization assay; SUDV, Sudan virus; IC_{50}, half-maximal inhibitory concentration; LOD, limit of detection; Ad26; adenoviral vector serotype 26; MVA, modified vaccinia Ankara; Ad35, adenoviral vector serotype 35; EBOV, Ebola virus; ELISA, enzyme-linked immunosorbent assay; EU, ELISA unit; MARV, Marburg virus; GP, glycoprotein; i.m. intramuscularly; vp, viral particle; TCID50, median tissue culture infectious dose; NHP, non-human primate.

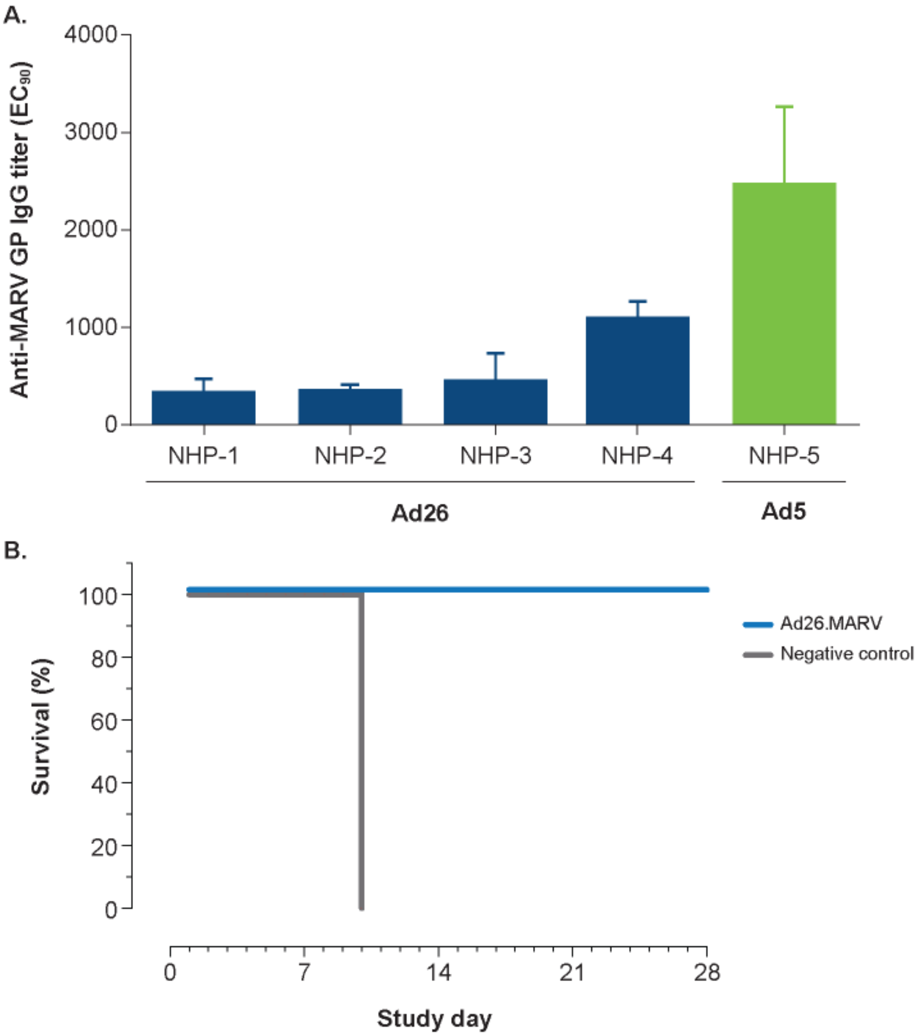

Supplementary Figure S2. Efficacy of a single administration of an Ad26 vector encoding the MARV GP. (**A**) Five cynomolgus macaques were immunized i.m. with a single dose of Ad26.MARV (n = 4) or Ad5.MARV (n = 1), and 1 animal was included as negative control. Adenovirus vaccines were given at 1 × 10^{11} vp, and 5 weeks after immunization the animals were challenged i.m. with 1000 pfu MARV Angola. Humoral responses were measured in serum 1 week prior to challenge. (**B**) Kaplan-Meier representation of survival. MARV, Marburg virus; GP, glycoprotein;

IgG, immunoglobulin G; EC_{90}, 90% effective concentration; NHP, non-human primate; Ad26, adenoviral vector serotype 26; Ad5, adenoviral vector serotype 5; vp, viral particles; pfu, plaque-forming units.

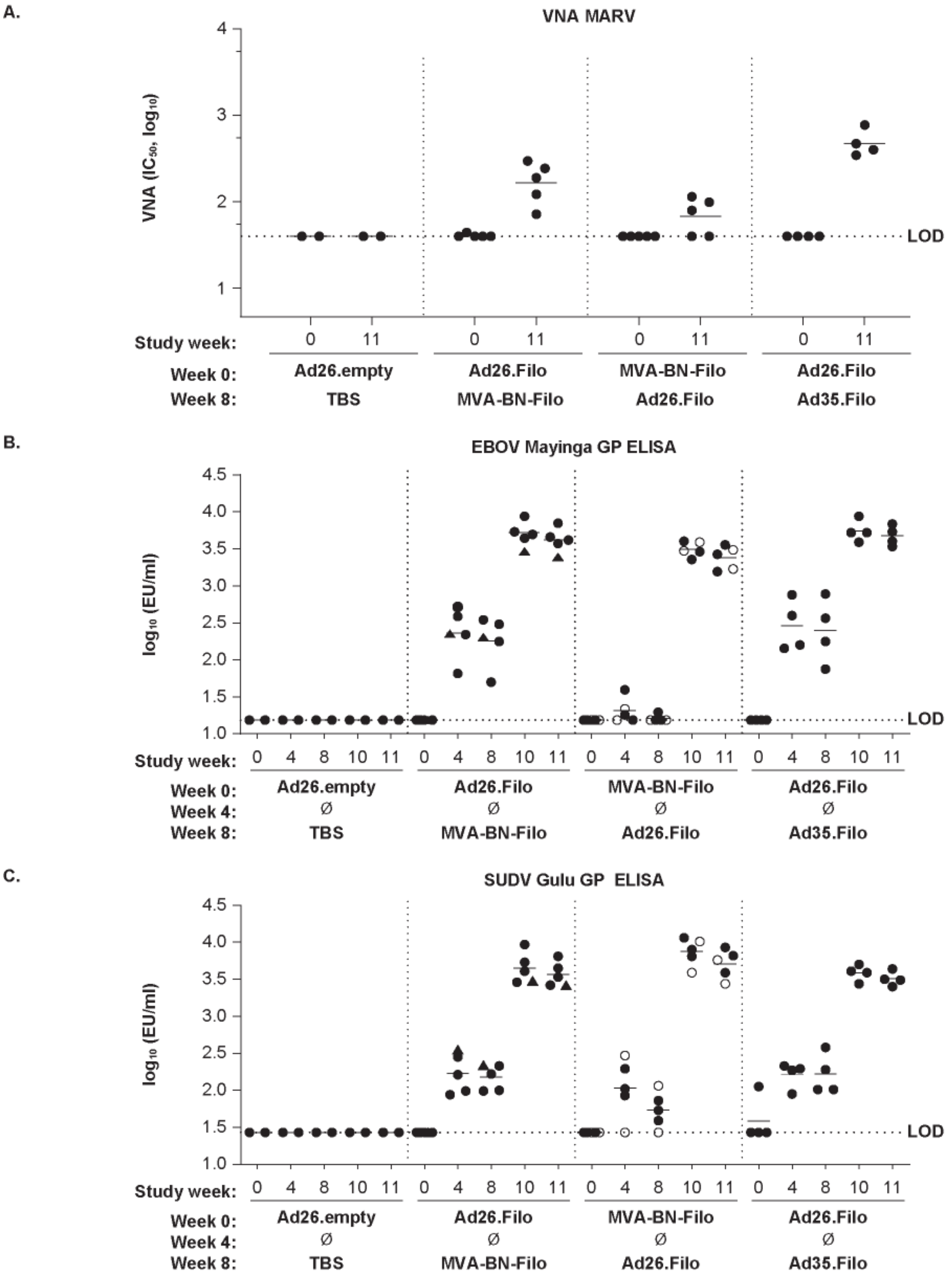

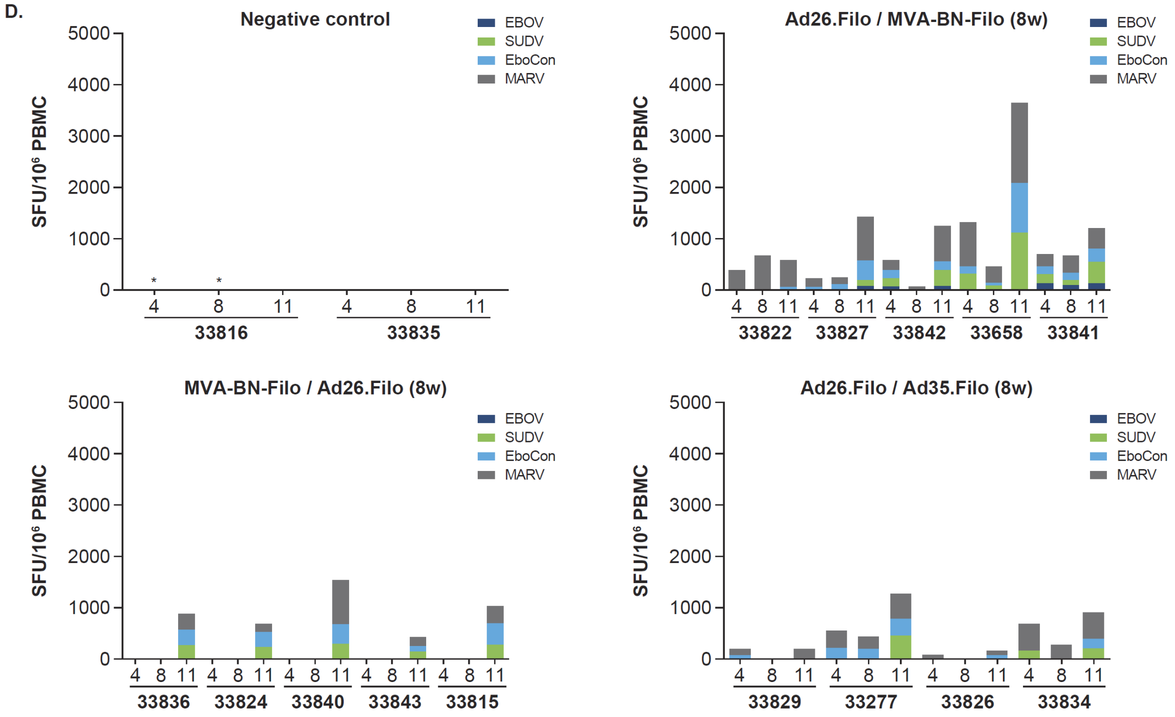

Supplementary Figure S3. Humoral and cellular immune responses. Fourteen cynomolgus macaques were immunized i.m. with a two-dose trivalent vaccine combination of Ad26.Filo, MVA-BN-Filo (n = 5), MVA-BN-Filo, Ad26.Filo (n = 5), or Ad26.Filo, Ad35.Filo (n = 4) with an 8-week interval; 2 cynomolgus macaques were included as negative controls and received Ad26 empty vector at week 0 and TBS at week 8. Adenovirus vaccines were given at 1.2×10^{11} vp (4×10^{10} vp/vector), and the MVA-BN-Filo vector at a dose of 5×10^{8} TCID50. (**A**) MARV Angola neutralization is expressed as IC_{50} titers on a log10 scale. The neutralization was measured in samples collected before dose 1 (t = 0) and 3 weeks post-dose 2 (week 11). The dotted line indicates lower limit of detection of the assay (LOD = 1.6 log10). Black bars indicate geometric mean of the titers within each group. (**B,C**) Humoral immune responses were measured over time by EBOV and SUDV GP–specific ELISA. Each symbol represents an individual NHP at the indicated time point, with the closed circles representing the non-symptomatic survivors, open circles the non-survivors, and triangles the symptomatic survivors. (**D**) Cellular immune responses were measured by IFN-γ ELISpot and are expressed as SFU per 10^6 PBMC against various peptide pools per animal at the indicated time points (responses were corrected for background responses). The EBOV peptide pool consists of two peptide pools, which are pools of EBOV Mayinga GP–specific peptides (51 and 49 peptides); SUDV consists of two pools containing 58 SUDV Gulu–specific peptides each; and MARV contains two peptide pools covering the complete MARV Angola GP sequence (43 and 44 peptides per pool). EboCon is a peptide pool containing 118 conserved peptides from EBOV GP, EBOV Ivory Coast GP, and SUDV Gulu GP. * Denotes high background. MARV, Marburg virus; VNA, virus neutralization assay; IC_{50}, half-maximal inhibitory concentration; LOD, limit of detection; Ad26, adenoviral vector serotype 26; MVA, modified vaccinia Ankara; Ad35, adenoviral vector serotype 35; EBOV, Ebola virus; GP, glycoprotein; ELISA, enzyme-linked immunosorbent assay; EU, ELISA unit; SUDV, Sudan virus; i.m., intramuscularly; TBS, Tris-buffered saline; vp, viral particle; TCID50, median tissue culture infectious dose; NHP, non-human primate; IFN-γ, interferon gamma; SFU, spot-forming units; PBMC, peripheral blood mononuclear cells.

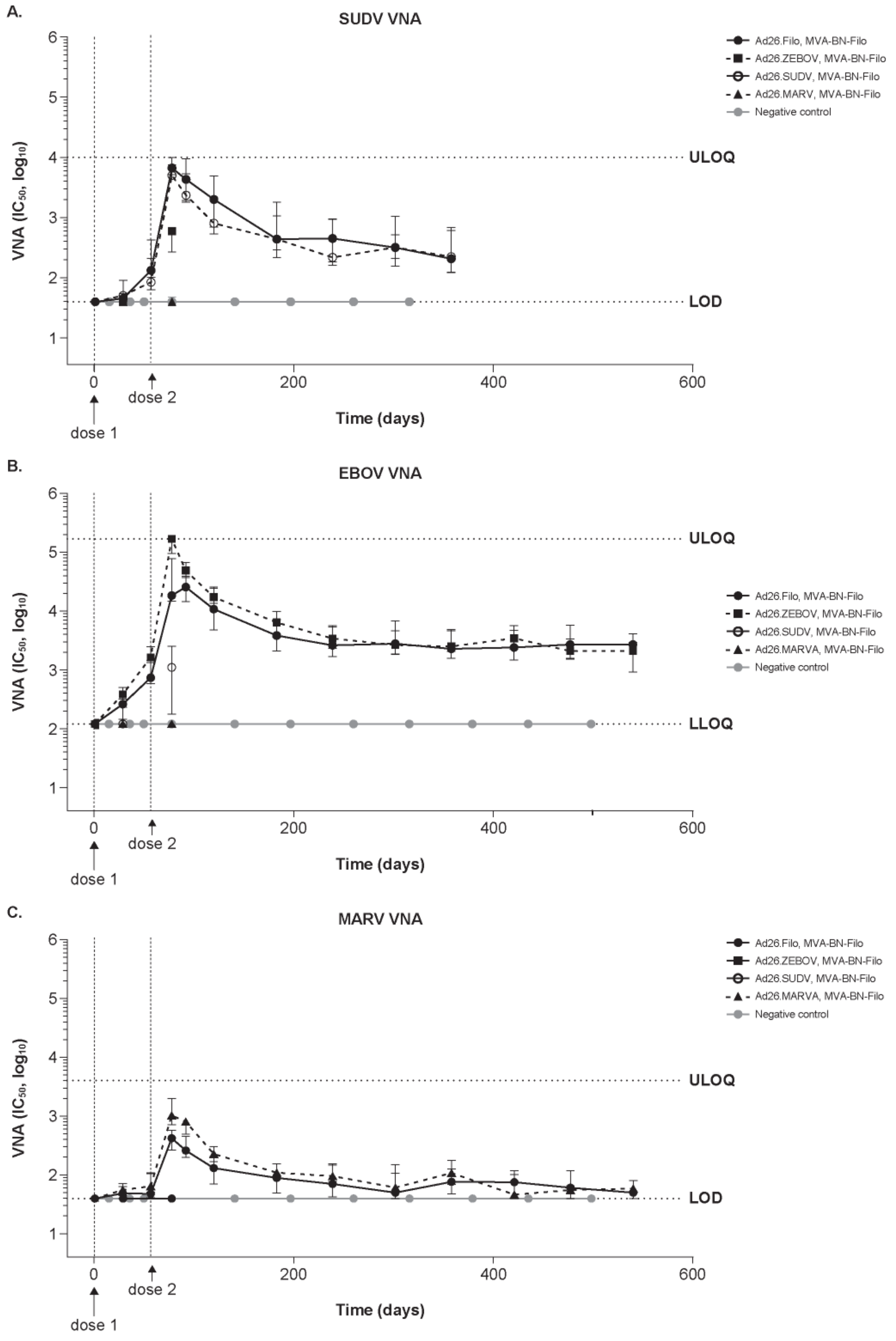

Supplementary Figure S4. Neutralizing GP-specific antibodies against SUDV, EBOV, and MARV. Cynomolgus macaques were immunized i.m. with a two-dose vaccine regimen, with an 8-week interval between dose 1 and dose

2.Virus neutralizing antibody responses were monitored up to day 358 (SUDV) or day 540 (MARV and EBOV). One group (n = 10) received the trivalent Ad26.Filo (1.2×10^{11} vp) followed by MVA-BN-Filo (5×10^{8} infU) as dose 2. Three other groups received a monovalent Ad26 (Ad26.EBOV, Ad26.SUDV, or Ad26.MARVA) at 4×10^{10} vp followed by MVA-BN-Filo (5×10^{8} infU) (n = 5 per group). A negative control group (n = 5) received two injections with saline 2 weeks apart. (**A**) SUDV, (**B**) EBOV Makona, and (**C**) MARV neutralization are expressed as IC_{50} titers on a log10 scale. Horizontal dotted lines indicate LLOQ or LOD of the assay and ULOQ. Vertical dotted lines indicate time of immunization. The points indicate median of the titers within the group, and whiskers show interquartile range. SUDV, Sudan virus; VNA, virus neutralization assay; IC_{50}, half-maximal inhibitory concentration; ULOQ, upper limit of quantification; LOD, limit of detection; Ad26, adenoviral vector serotype 26; MVA, modified vaccinia Ankara; EBOV, Ebola virus; LLOQ, lower limit of quantification; MARV, Marburg virus; i.m., intramuscularly; vp, viral particle; infU, infectious unit.

Reference

1. Callendret, B.; Vellinga, J.; Wunderlich, K.; Rodriguez, A.; Steigerwald, R.; Dirmeier, U.; Cheminay, C.; Volkmann, A.; Brasel, T.; Carrion, R., et al. A prophylactic multivalent vaccine against different filovirus species is immunogenic and provides protection from lethal infections with Ebolavirus and Marburgvirus species in non-human primates. *PLoS ONE* **2018**, *13*, e0192312; https://doi.org/10.1371/journal.pone.0192312.
